# Ultraviolet Detectors Based on Wide Bandgap Semiconductor Nanowire: A Review

**DOI:** 10.3390/s18072072

**Published:** 2018-06-28

**Authors:** Yanan Zou, Yue Zhang, Yongming Hu, Haoshuang Gu

**Affiliations:** 1 School of Science, Jilin Institute of Chemical Technology, Jilin 132022, China; zyndoudou@163.com; 2 Hubei Key Laboratory of Ferro & Piezoelectric Materials and Devices, Faculty of Physics and Electronic Sciences, Hubei University, Wuhan 430062, China; zhangyuehb@outlook.com

**Keywords:** UV detectors, wide bandgap semiconductor, nanowire

## Abstract

Ultraviolet (UV) detectors have attracted considerable attention in the past decade due to their extensive applications in the civil and military fields. Wide bandgap semiconductor-based UV detectors can detect UV light effectively, and nanowire structures can greatly improve the sensitivity of sensors with many quantum effects. This review summarizes recent developments in the classification and principles of UV detectors, i.e., photoconductive type, Schottky barrier type, metal-semiconductor-metal (MSM) type, *p-n* junction type and *p-i-n* junction type. The current state of the art in wide bandgap semiconductor materials suitable for producing nanowires for use in UV detectors, i.e., metallic oxide, III-nitride and SiC, during the last five years is also summarized. Finally, novel types of UV detectors such as hybrid nanostructure detectors, self-powered detectors and flexible detectors are introduced.

## 1. Introduction

Ultraviolet (UV) detectors have received considerable attention due to their important applications in the military and civilian realms. They are used in defense warning systems, UV communication, space science, environmental monitoring, industrial production, medicine and healthcare [1,2,3,4].

**Figure 1:**
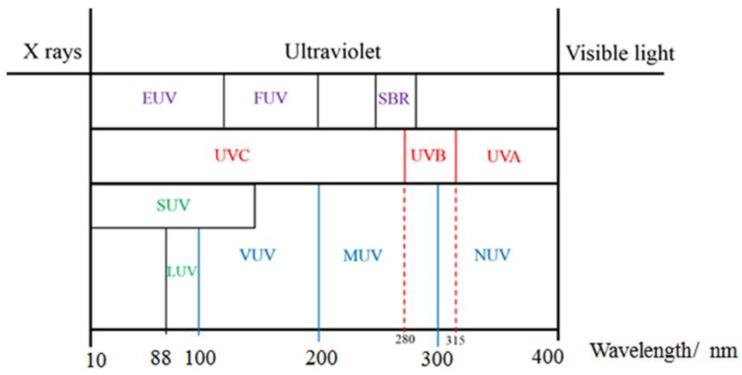
The classification of UV light.

UV light is a general term for a specific band of electromagnetic radiation with a wavelength range of 100–400 nm and energy distribution range of 3.1–12.4 eV. Figure 1 shows the common classification of UV light, which can be divided into the following bands: UVA (400–315 nm), UVB (315–280 nm), UVC (280–200 nm), and VUV (200–10 nm) [5]. When solar UV radiation passes through the ozone layer, the 240–280 nm wavelengths are strongly absorbed. Hence, such radiation is almost non-existent in the atmosphere near the ground, and comprises the so-called solar-blind region (SBR) [6]. In the SBR, it is easy to detect a target signal, false alarms are rarely produced, and there is little background interference. For this reason, UV detection has an advantage over infrared and laser detection technology. UV radiation in the wavelength range of 300–400 nm has a high penetration rate and can reach the ground, so this radiation band is called the UV window to the atmosphere.

Wide bandgap semiconductors have many useful characteristics, such as low permittivity, high breakdown electric-fields, good thermal conductivity, high electron saturation rates and radiation resistance. Hence, they can work at high temperatures, and are also ideal for developing semiconductor devices with high frequency and power, and high temperature and radiation resistance [7]. Taking III-group nitrides as an example, in terms of optical properties, their optical band gap can vary continuously from 0.77 eV (InN) to 6.28 eV (AlN), completely covering the infrared to ultraviolet band [8]. Since wide bandgap semiconductors can absorb and radiate high-energy photons, they are the most suitable candidates for fabricating blue, green and other short wavelength light emitting diodes (LEDs), semiconductor lasers and UV detector devices [9,10,11,12], which are widely used in the fields of optoelectronics and microelectronics.

In 2002, Yang and coworkers used a ZnO single nanowire for a UV detector and obtained an excellent UV response, setting off a stream of research into the use of one-dimensional (1D) nanomaterials for UV detection [13]. Nanowires are considered a promising building block for LEDs and sensors [14]. A large specific surface area and the presence of deep level surface trap states in nanowires greatly prolongs the photocarrier lifetime. Meanwhile, the reduced dimensionality of the active area in nanowire devices shortens the transit time of the carrier, resulting in substantial photoconductive gain [15,16]. In this way, photodetectors based on 1D nanowires can have excellent performance with superior sensitivity, high quantum efficiency, and fast response speed [16]. Thus, wide bandgap semiconductor nanowires are thought to be promising materials for use in optoelectronic devices [17,18,19,20,21,22,23], especially UV detectors or sensors [24,25,26,27,28].

There are few comprehensive reviews focusing on wide bandgap nanowire-synthesized UV detectors. In this article, wide bandgap semiconductor nanowire-based UV detectors are discussed in detail. First, a visual classification of UV detectors is presented. The mechanism imposed on each type of detector, as well as the inherent advantages and disadvantages of those detectors, are discussed. Recent research into materials suitable for producing nanowires for use in UV detectors and new types of UV detectors is also summarized.

## 2. UV Detectors

**Figure 2:**
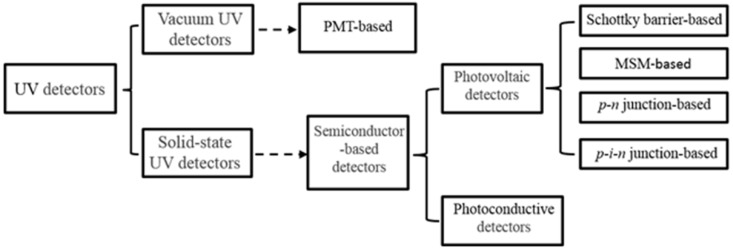
Classification of UV detectors.

The classification of several typical kinds of UV detector will be introduced as follows (Figure 2): in general, UV detectors fall into two categories: vacuum and solid-state [5,29]. Vacuum UV detectors are based on different kinds of photomultiplier tubes (PMTs) and their derived imaging devices. The technology of PMT-based UV detectors is relatively mature, and it can achieve large area detection, high gain and rapid response. Unfortunately, heavy weight, low efficiency, high power consumption, fragility and high-pressure operations limit the application of vacuum UV detectors [29,30].

**Figure 3:**
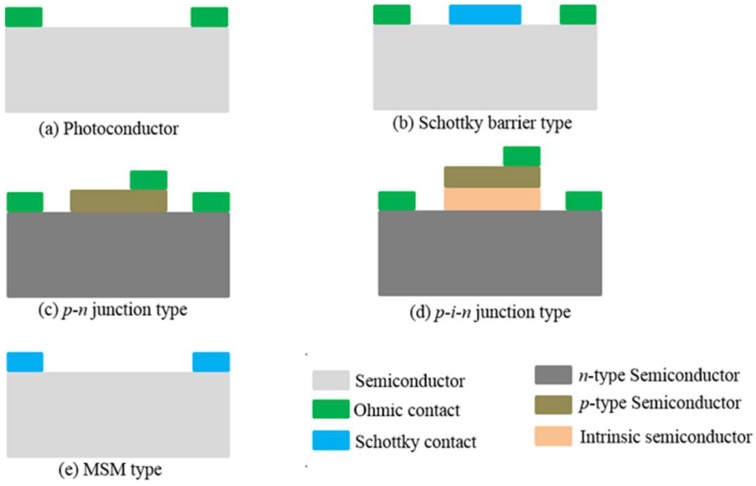
Schematic structure of the different types of wide bandgap semiconductor UV detector.

Solid-state UV detectors are mainly based on semiconductor materials. This article will focus on wide bandgap semiconductor nanowire materials. According to the various basic working principles, wide bandgap semiconductor UV detectors can be divided into photoconductive UV detectors and photovoltaic UV detectors [29]. Photovoltaic UV detectors also can be separated into the Schottky barrier type, metal-semiconductor-metal (MSM) type, *p-n* junction type, and *p-i-n* junction type. Those common schematic structures are shown in Figure 3 [31,32]. In this section, a brief description of the working mechanisms and advantages and disadvantages of each type of UV detector will be given.

### 2.1. Photoconductive Detectors

**Figure 4:**
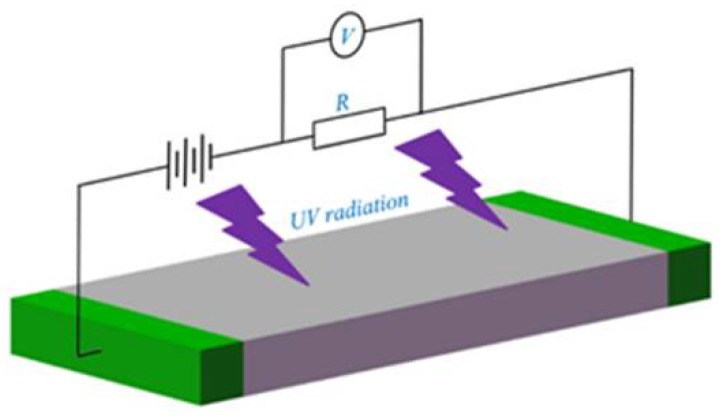
The operation of a photoconductor.

Photoconductive detectors, also named photoconductors, are a kind of light detector that work based on the photoconductive effect. A piece of semiconductor material and two Ohmic contacts can make up a photoconductive UV detector, as shown in Figure 3a. The operation of a photoconductor is shown in Figure 4. When a photon’s energy hυ is greater than the band-gap energy *E_g_*, it will be absorbed to produce an electron-hole pair, consequently changing the electrical conductivity of the semiconductor. Then, an external circuit detects the variation of electric conductivity [33,34]. Takahashi et al. [35] designed nitrogen-doped ZnO single crystals for use in photoconductive UV sensors. Nitrogen-doping improved significantly the photocurrent, photosensitivity, UV-visible rejection ratio, and time response of ZnO-based UV sensors.

Photoconductive UV detectors have simple structures, easy process control and high internal gain, but their slow response speed and large dark current remain challenging.

### 2.2. Schottky Detectors

**Figure 5:**
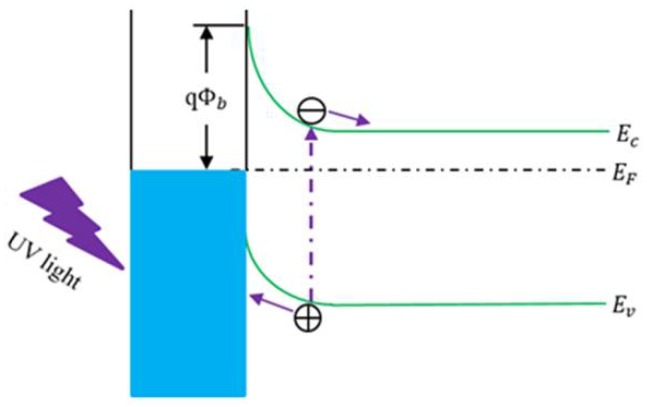
The schematic operation of the mental-(*n*-type) Schottky photoconductor.

The Schottky structure is a common structure used in wide bandgap semiconductor UV detectors. It is a Schottky diode consisting of a semitransparent Schottky contact and an Ohmic contact [36]. The schematic structure of a Schottky photodetector is shown in Figure 3b and its operation is shown in Figure 5. The metal/semiconductor junctions exhibit rectifying behavior. The rectifying property of the metal-semiconductor contact arises from the presence of an electrostatic barrier between the metal and the semiconductor, which is attributed to the difference (*q*$\Phi_{b}$) in the work functions of the metal and semiconductor [37]. Zhao et al. [38] fabricated an asymmetric Schottky barrier UV detector based on an Ag/ZnO/Au structure, which exhibited excellent performance. The photocurrent generated from the device under UV illumination was about 120 nA without applying any external bias, and it also had a fast switching time of less than 30 ms. This phenomenon was explained by using Schottky barrier and O_2_ adsorption-desorption theories.

Schottky UV detectors have many advantages, such as high responsivity, high quantum efficiency, low dark current, short response time, and possible zero-bias operations. However, for Schottky contact, the incident light should pass through the metal electrode to interact with the semiconductor (photoactive layer), so metal electrode is usually made into a very thin semi-transparent layer, while high absorption coefficient of metal electrode is a problem in Schottky detector structure. Besides, surface state effect, and shallow metal-semiconductor contact, which has restricted the development of Schottky structure UV detectors to a certain extent [36].

### 2.3. p-n and p-i-n Junction Detectors

A *p-n* junction detector is a *p-n* junction diode specifically manufactured to ensure that the detected light can penetrate into the nearby metallurgical junction. Its structure is schematically shown in Figure 3c. When light of the proper frequency irradiates the active region of the detector, a photon-generated carrier forms a photocurrent in the external circuit under an electric field, being no gain for the normal photovoltaic work mode. When forward bias is applied in the *p-n* junction, the dark current is far greater than the photocurrent and the detector is unable to work due to unilateral conductivity characteristics. Under reverse bias, the dark current is suppressed, and moreover, the carrier transit time and diode capacitance are reduced, which are benefit to the improvement of sensitivity. [39,40,41,42]. A ZnO microwire *p*-*n* homojunction UV photodetector with high efficiency and high wavelength selectivity was demonstrated by Shi et al. [42]. The photodetector was formed by a single Sb-doped ZnO microwire and a single undoped ZnO microwire.

To improve the sensitivity and response speed of devices, an intrinsic *i* layer can be placed between the *p* and *n* layers to increase the width of the depletion layer, as shown in Figure 3d. In general, *p-i-n* photodiodes work at zero bias or reverse bias (photoelectric diode mode), so that the difference between the photocurrent and dark current can reach the maximum and improve the sensitivity. With increasing reverse bias, the depletion region will be widened and the junction capacitance will be reduced, enhancing not only responsivity, but also the response speed [29]. In order to improve the performance of a *p-i-n* device, a key factor is to increase the light transmission rate, which requires a structure designed so that light is absorbed in the depletion region. Therefore, selecting a translucent metal as the Ohmic contact and decreasing the thickness of the *p* layer are beneficial [43]. Bugallo et al. reported a visible-blind UV photodetector based on *p-i-n* GaN nanowire ensembles on a Si substrate [44]. The detector peak responsivity, the UV-to-visible rejection ratio, and operation speed were quite good. Their proposed technology of *p-i-n* nanowires on Si substrates opens a path for the commercialization of efficient visible-blind detectors.

**Figure 6:**
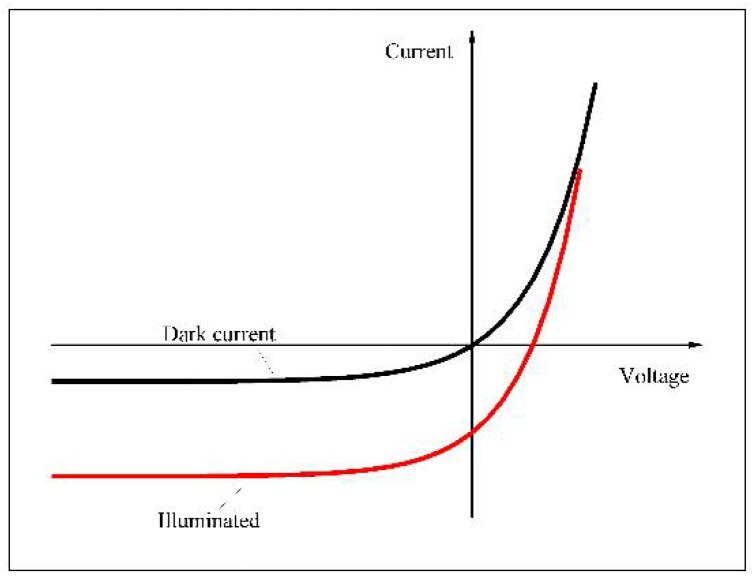
The *I-V* characteristic of a *p-n* photodiode.

The *I-V* characteristic of a *p-n* photodiode is shown in Figure 6. The total current can be expressed using the following equation [32]:

(1) $$I\left(V\right)=I_{s}\left[exp\left(\frac{eV}{nkT}\right)-1\right]-eG$$

where $I_{s}$ is the saturation current, *V* is the applied voltage, *n* is the ideality factor, *k* is Boltzmann’s constant, *T* is absolute temperature and *G* is the generation rate. $I_{s}\left[exp\left(\frac{eV}{nkT}\right)-1\right]$ and −eG correspond to the dark current and photocurrent, respectively.

The *p-n* and *p-i-n* photodiodes have the advantages of fast response speed, high impedance, low dark current, low or zero bias operation, capability of high-frequency operation, and compatibility of the fabrication technology with planar-processing techniques. However, their time response is usually limited by the behavior of *p*-dopants, which can also deteriorate the spectral response.

### 2.4. MSM Detectors

**Figure 7:**
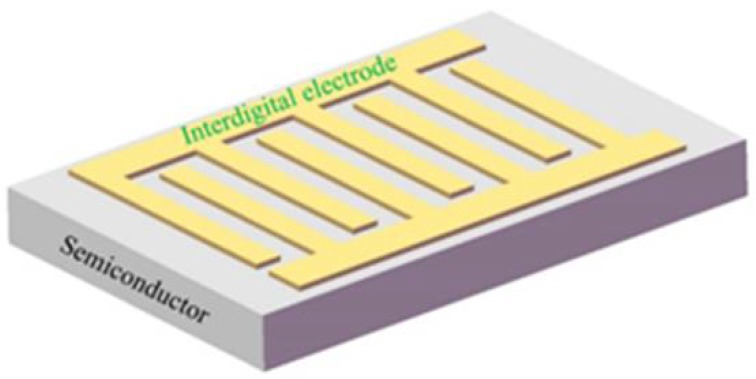
The schematic structure of an inter-digital electrode.

MSM detectors are made up of double “back-to-back” semiconductor Schottky barriers by using an inter-digitated electrode with planar linear on top of an active light collection region, as shown in Figure 7 [45,46]. Figure 3e shows its schematic structure. An MSM photodetector cannot operate at a zero bias. When under the DC bias, one of the Schottky barriers will be forward biased and the other will be reverse biased, so the dark current is quite small. Because of the low capacitance per unit area and limited transit time, MSM photodiodes also have an intrinsic rapid response ability. These devices are linear with optical power, and present a UV/visible contrast of 10^4^. Menzel et al. [47] reported a simple fabrication of a ZnO-nanowire-based device with applications in UV detectors, pH sensors, and temperature sensors. This nanowire sensor structure provides Schottky barriers due to the MSM structure.

MSM detectors have a high and fast response, are little affected by bias, and have simple manufacturing processes, low cost and easy monolithic integration. However, because the metallization for the electrodes shadows the active light collecting region, it also exhibits low gain and spectral response.

### 2.5. Important Parameters of UV Detector Devices

Usually, we will choose a variety of parameters to characterize a device. Hence, some important parameters of UV detector devices are summarized as follows:

1. Cut-off wavelength: The longest wavelength ($\lambda_{0}$) that the UV detector can detect, referring to Equation (2) [48]. It can be measured by absorption and a transmittance spectrum, where *h* is Planck’s constant, *c* is the speed of light and $E_{g}$ is the band gap:
  (2) $$\lambda_{0}=\frac{hc}{E_{g}}$$
2. Photocurrent: The current formed in the external circuit. It can be tested at different biases, *I-V* measurement under UV radiation, visible light and dark conditions.
3. Dark current: The current which remains in the detector without UV radiation. Dark current is equivalent to a noise source and will weaken the signal-to-noise ratio.
4. Time response: When the UV light turns on or off, the required time of the output value rises to become stable or decreases to the value before irradiation, accordingly. This implies the sensitivity of the device.
5. Quantum efficiency: Every incident UV photon will try to produce an electron-hole; however, the number of created electron-hole pairs is usually less than the number of photons. This represents the quantum efficiency, which can be calculated by [49]:
  (3) $$\eta \left(\lambda \right)=R_{\lambda }\frac{hc}{q\lambda }$$
  where $R_{\lambda }$ is the measured responsivity at incident light wavelength λ, and *q* is the electron charge.

In conclusion, high-performance UV detectors require high sensitivity, high stability and high operational speed [5]. Considering the key requirements listed above, solid-state UV detectors are more attractive than vacuum ones because they offer higher sensitivity, better linearity and higher operational speed. On the second level of Figure 2, semiconductor-based devices are chosen for their high stability, outstanding linearity, compactness and low weight. On the third level, photovoltaic detectors provide advantages both in terms of sensitivity and operational speed compared to photoconductive detectors.

## 3. Materials for UV Detectors

High performance, quantum efficiency, response speed, and signal-to-noise ratio are the most important features of UV detectors. The performance of a UV detector is mainly dependent on the inherent characteristics of the semiconductor materials used. The ability to transform optical signals into electrical signals is determined by a complex process of electron-hole pair generation, transportation, and recombination within the semiconductor materials. Therefore, in the next section, we will summarize wide bandgap semiconductor materials including metallic oxide, III-nitride, and SiC. Corresponding nanowire-structured UV detectors are discussed in following sections.

### 3.1. Metal Oxides

Metal oxide materials are the focus of current research efforts since they are the commonest minerals in the earth. 1D metal oxide nanostructures have been extensively investigated for the fabrication of nanoscale photodetectors [50]. Common materials include ZnO, TiO_2_, and SnO_2_; thus, in this section, we comprehensively review the above materials and introduce other metal oxides, including VO_2_, β-Ga_2_O_3_, and In_2_O_3_. Ternary oxides are also described.

#### 3.1.1. ZnO

ZnO is a direct band gap II-VI-group semiconductor material. It has strong radiation hardness, high chemical stability and is abundant. It has ionicity between covalent bonding semiconductors and ionic bonding semiconductors. At room temperature (300 K) and atmospheric pressure, its band gap width is *E_g_* = 3.365 ± 0.005 eV. Changes in temperature and pressure influence the width of the band gap. In addition, the exciton binding of ZnO is 60 meV, far higher than thermal ionization (26 meV) under room temperature. Therefore, its excitons can remain stable at room temperature, and realize effective excitation and emission at room temperature, make them suitable for shortwave photoelectric devices [12,51,52,53,54,55,56] and a very good candidate for photonics devices working in the UV range [15,57,58,59,60]. Various methods have been used in attempts to synthesize ZnO nanomaterials for use in next-generation photoelectric devices. These materials include ZnO nanowire [61], films [62], nanorods [63,64], nanoparticles [65,66], nanobelts [67], nanowalls [68], and other hybrid structures [69].

**Figure 8:**
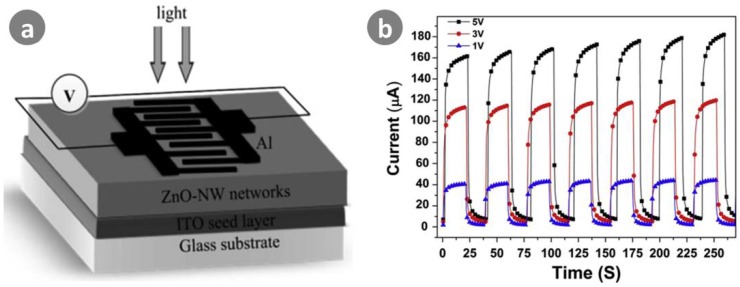
(**a**) Schematic of fabricated ZnO-nanowire networks UV photodetector; (**b**) Photoresponse of ZnO-nanowires UV-photodetector at various bias voltages under pulsed UV light with interval of 20 s. Ref. [71]. Copyright 2016, Elsevier Science EV.

ZnO nanowires have much greater photo-electronic sensitivity than ZnO film under UV radiation due to the nanowire carrier transport channel and much higher exciton bonding energy [15,63]. This results in ZnO nanowires being efficient in generating electron-hole pairs during UV illumination. In recent years, ZnO nanowires have become one of the most promising functional components for UV detectors [14,15,70,71,72,73,74,75,76]. Mallampati et al. [70] synthesized ZnO nanowires by a vapor phase transport process. Their MSM device had a responsivity in the order of 10^5^ A/W, corresponding to extremely high photoconductive gain in the order of 10^6^. Alsultany et al. [71] fabricated the first MSM UV photodetector based on catalyst-free growth of ZnO nanowire networks on indium tin oxideseeds/glass substrates by a thermal evaporation method, as shown in Figure 8. Upon exposure to 365 nm light (1.5 mW/cm^2^) at a 5 V bias, their device showed sensitivity of 2.32 × 10^3^%. In addition, the photocurrent was 1.79 × 10^−4^ A, and the internal gain of the photodetector was 24.2. The response and recovery times were calculated as 3.9 and 2.6 s, respectively.

Li et al. [72] found that the response speed and photo-to-dark current ratio of ZnO nanowire UV photodetectors can be optimized by tuning the entanglement and density of ZnO nanowires grown on SiO_2_ pillars through a catalyst-free chemical vapor deposition process. Their ZnO nanowire UV photodetector exhibited a rise time of 0.45 s and a decay time of 0.06 s, with a high photocurrent to dark current ratio of 10^2^.

**Figure 9:**
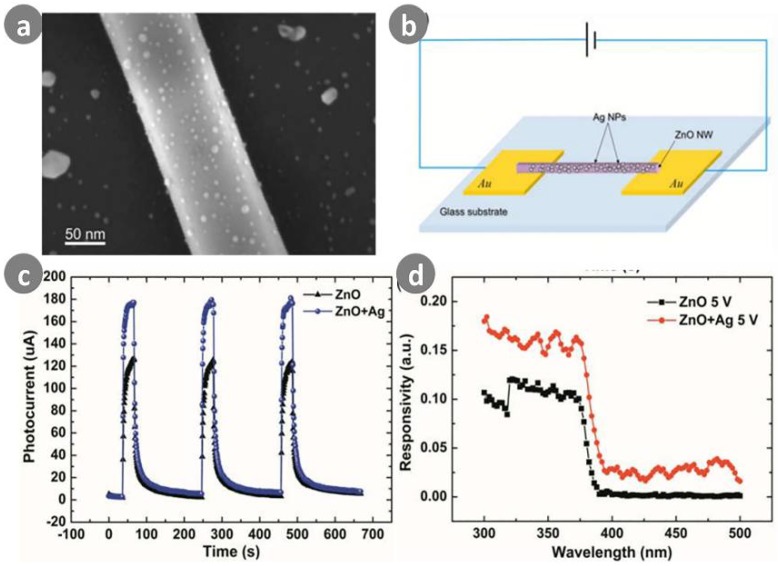
(**a**) Ag nanoparticles on the surface of ZnO nanowire; (**b**) Schematic image of the complete device with double Schottky barrier contacts; (**c**) Time dependence of the photocurrent under multiple on/off cycles; (**d**) Spectral responsivity of the devices with two type of conducting channel at 5 V bias. Ref. [73] Copyright 2016, Royal Society of Chemistry.

A large number of studies have investigated improvements to sensitivity and reset time. Nanophotodetectors combined with metal particles can induce plasmonics enhancement. For example, Zhao et al. [73] fabricated a ZnO nanowire-based UV photodetector with double Schottky barrier contacts using electric field guided assembly technology. Reasonable improvements were achieved by applying uniform-sized Ag nanoparticles onto the ZnO nanowire surfaces. As shown in Figure 9, the responsivity and photoconduction gain of the decorated device reached 4.91 × 10^6^ A/W and 1.67 × 10^7^, respectively. The enhanced performance was associated with enhanced surface trap states and localized surface plasmon resonance effects from the uniform Ag nanoparticles. The nanoparticles played an important role in the localized gating effect, inducing dark current change in the nanoscale conductive channel.

Graphene has attracted much attention in optoelectronic devices because of its excellent carrier transport mobility, high electrical conductivity, and mechanical flexibility [74]. UV photodetectors based on vertically-aligned ZnO micro/nanowires on graphene were prepared by Liu et al. [75], which showed a high responsivity of 1.62 A/W. Boruah et al. [76] grew a hybrid structure of ZnO nanowires on graphene foam for UV detection. Excellent photoresponse was obtained, with response and recovery times of 9.5 and 38 s, respectively, and an external quantum efficiency of 2490.8%.

#### 3.1.2. TiO_2_

Titanium dioxide (TiO_2_) is one of the most extensively researched materials owing to its excellent chemical stability, non-toxicity, thermal stability and low-cost. TiO_2_ is an n-type semiconductor with a wide band gap ranging from 3.0 to 3.2 eV for rutile and anatase, respectively, and has great potential for application to UV detection. Different morphologies of nanostructured TiO_2_ have been reported for use in UV detectors, such as TiO_2_ films [77,78], TiO_2_ nanorods [79,80], and TiO_2_ nanowires; hence, TiO_2_ is a promising candidate due to its unique optical and electronic properties.

Liu et al. [81] synthesized TiO_2_ nanowire arrays (TNAs) with different morphologies on transparent conductive tin-doped indium oxide (ITO) and fluorine-doped tin oxide (FTO). They assembled back-incident array TNA-based UV detectors and demonstrated that single crystallographic orientation TNAs on FTO performed well. Similarly, Zhang et al. [82] synthesized vertically-oriented TiO_2_ nanowires on FTO-coated glass and fabricated a hybrid UV detector by spin-coating a thin layer of poly (9,9-dihexylfluorene) onto the TiO_2_ array. The dark current of the detector was as low as 1.9 nA and a photoresponse peak of 568 mA/W was obtained.

**Figure 10:**
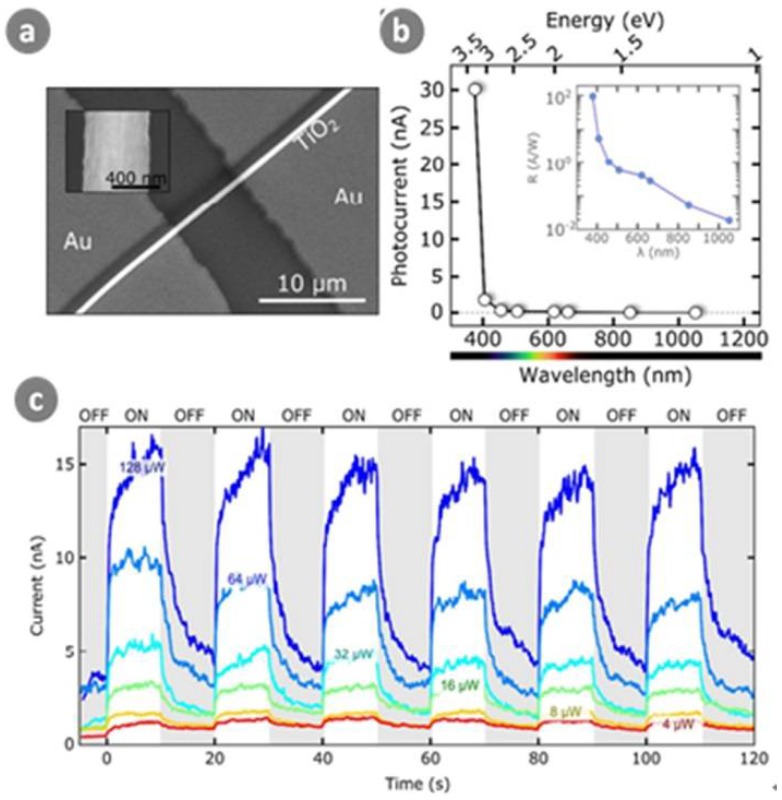
(**a**) SEM image a TiO_2_ fiber photodetector. Inset: zoom of the fiber; (**b**) Photocurrent of the device shown in (**a**) as a function of the LED wavelength (*P* = 2 µW, *V*_b_ = 10 V). Inset: responsivity as a function of the LED wavelength; (**c**) Time response of the photodetector. In order to highlight the photocurrent, the dark current has been set to 0. The measured rise time is ~2.5 s and the fall time is ~10 s. Ref. [84] Copyright 2016, Royal Society of Chemistry.

Similarly, Chen et al. [83] prepared a photodetector based on TiO_2_ nanowires by an inkjet printing method. The printed photodetector was highly transparent with a visible transmittance of 85% and showed a low dark current of 10^−12^ A with a high on/off ratio of 2000. Under a bias voltage of 2 V, a fast rise time of 0.4 s was achieved, with a recovery time of 0.1 s. Molina-Mendoza et al. [84] presented UV photodetector devices based on individual electro-spun TiO_2_ nanofibers transferred onto pre-patterned electrodes. As shown in Figure 10, the fabricated devices demonstrated an outstanding UV photoresponse of ~90 A/W and a response time of ~5 s.

#### 3.1.3. SnO_2_

Tin dioxide (SnO_2_) is an n-type oxide with a wide bandgap (*E_g_*) of around 3.6 eV at room temperature. SnO_2_ is a useful material that can be applied as a transparent conductive oxide because of its unique optical properties and chemical stability. It is also a potential material for use in UV photodetectors. Nanostructures of SnO_2_ can be fabricated as nanowhiskers [85], nanobelts [86], nanorods [87,88,89], and nanowires [90].

Hu et al. [91] successfully produced thin SnO_2_ nanowires with a uniform diameter. Then, an individual SnO_2_ nanowire-based UV detector was constructed, which exhibited excellent light selectivity and stability. More importantly, a very promising characteristic of the device is its ultrahigh EQE value of 1.32 × 10^7^. Via CuInS quantum dots decorated onto a single SnO_2_ nanowire, Lu et al. [92] successfully fabricated an ultra-high sensitivity photodetector. The gain value reached 2.5 × 10^5^ under 325 nm illumination. Similarly, Lupan et al. [93] synthesized single crystalline SnO_2_ nanowires with Zn_2_SnO_4_ dots functionalized surface. The individual SnO_2_: Zn_2_SnO_4_ nanowire based UV photodetector exhibited photoconductive performance in terms of high response to the 375 nm ultraviolet light irradiation, ultra-fast response and recovery time at different temperatures (25–300 K).

**Figure 11:**
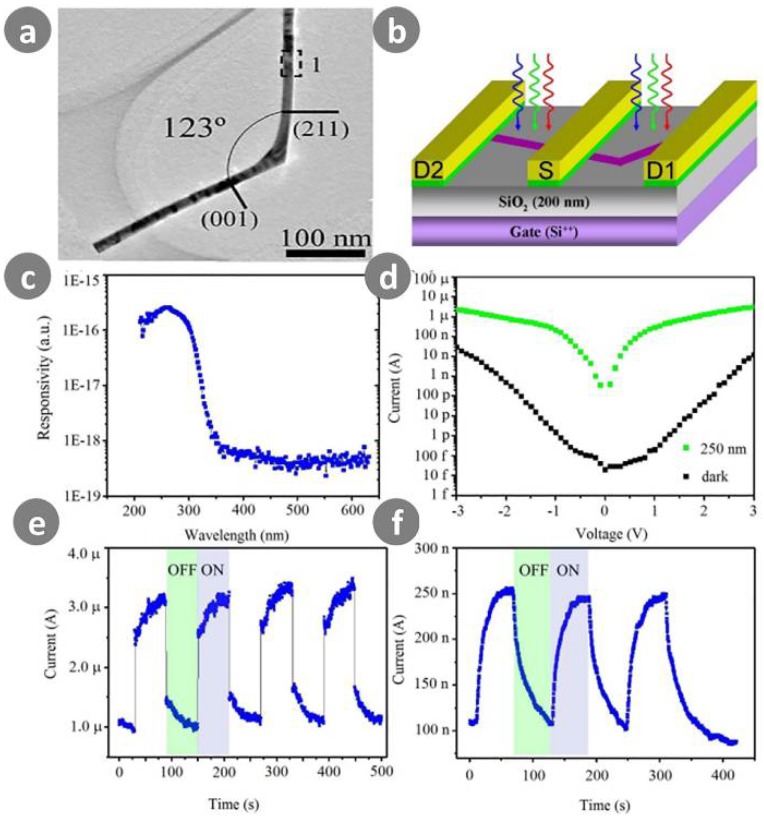
(**a**) Typical TEM image of kinked SnO_2_ nanowire, demonstrating the measured angle of 123° between the two arms; (**b**) A sketch for the device composed of straight and kinked nanowire devices from a single crystalline SnO_2_ nanowire; (**c**) Spectral photoresponse measured on the kinked nanowire device (Source-Drain1) at a bias of 3 V; (**d**) The comparison of photocurrent on the kinked nanowire device under 250-nm-wavelength incident light illumination (upper, green) and dark (lower, black); (**e**,**f**) On/off switching test under 250 nm incident light illumination at a bias of 3 V for the kinked nanowire device and straight nanowire device (Source-Drain2), respectively. Ref. [94] Copyright 2015, Royal Society of Chemistry.

Gan et al. [94] were the first to emphasize the important role of nanowire geometry in the improvement of photodetector performance, based on their study of a single crystalline kinked SnO_2_ nanowire. They demonstrated novel and simple geometry-induced high performance in a UV photodetector, as shown in Figure 11. The kinked SnO_2_ nanowire-based photodetector had a clear advantage over a straight SnO_2_ nanowire-based photodetector, both in photocurrent and photoresponse speed. Surprisingly, the photoresponsivity and EQE of the kinked SnO_2_ nanowire-based photodetector were ultrahigh, at 1.2 × 10^7^ A/W and 6.0 × 10^9^%, respectively, at most. A geometrical factor—the kinked nanowire structure—is mostly responsible for such improvement.

#### 3.1.4. Others

In addition to the metal oxide materials mentioned above, there are other metal oxide nanowires that have been studied. Wu et al. [95] demonstrated a single microwire photodetector made using a VO_2_ microwire, which exhibited high responsivity and external quantum efficiency under varying light intensities. Under illumination of 1 μW/cm^2^, the critical UV photodetector parameters of responsivity, external quantum efficiency, and detectivity, were observed as 7069 A/W, 2.4 × 10^10^%, and 1.5 × 10^14^ Jones, respectively, at a constant low bias of 4 V. Du et al. [96] investigated a high-performance deep UV photodetector based on a *β*-Ga_2_O_3_ nanowire network. Its high sensitivity, superior selectivity, ultrafast response speed and simple fabrication technology show that *β*-Ga_2_O_3_ nanowire networks have potential for application in solar-blind photodetectors. The photoconductor exhibited high responsivity in the 200–250 nm range with a sharp cutoff at 270 nm. Meng et al. [97] designed a novel photodetector based on square In_2_O_3_ nanowires with exposed four {001} facets. The photodetector delivered excellent optoelectronic performance, including excellent repeatability, fast response speed, high spectral responsivity, and external quantum efficiency. The *R*_λ_ and EQE values were as high as 4.8 × 10^6^ A/W and 1.46 × 10^9^%, respectively.

Ternary oxide nanowires are chemically and thermally stable and are superior for deep UV detection due to their large bandgap and high wavelength selectivity. Liu et al. [98] successfully fabricated a new UV-A photodetector based on a K_2_Nb_8_O_21_ nanowire. The single-nanowire photodetectors showed remarkable sensitivity and wavelength selectivity with respect to UV-A light. Furthermore, the photodetectors exhibited rapid response, a high discrimination ratio, robust stability, and a strong dependence of photocurrent on light intensity. Lou et al. [99] prepared InGaO_3_(ZnO) superlattice nanowire devices on a SiO_2_/Si substrate. They exhibited excellent sensitivity to UV irradiation, with a spectral responsivity of 5.3 × 10^4^ A/W, high external quantum efficiency of 1.9 × 10^7^, and fast response speed of 0.3 s. They also fabricated a photodetector using a single ZnGa_2_O_4_ nanowire on a SiO_2_/Si substrate [100]. This photodetector showed good sensitivity to UV light, and excellent properties such as high *I*_on_/*I*_off_ and external quantum efficiency values, and good reversibility. Zhou et al. [101] prepared a high-performance solar-blind DUV photodetector using individual chemical vapor deposition (CVD)-fabricated Zn_2_GeO_4_ nanowires. The Zn_2_GeO_4_ nanowire-based solar-blind DUV photodetector demonstrated outstanding sensing performance, photoresponsivity of 5.11 × 10^3^ A/W, external quantum efficiency of 2.45 × 10^6^%, detectivity of ≈2.91 × 10^11^ Jones, and τ_rise_ ≈ 10 ms and τ_decay_ ≈ 13 ms.

### 3.2. III-Nitride Semiconductors

III-nitride compound semiconductors are composed of group III elements (aluminum, gallium, or indium) and the group V element (nitrogen) [2,102,103]. These include GaN, AlN, InN, and multielement alloys composed of these compounds, such as InGaN, AlGaN, and InAlGaN. III–V group materials with a wurtzite structure are direct band gap materials. By changing the alloy composition, the width of their forbidden band can be varied continuously. For AlGaN, the forbidden band can continuously change from 3.4 eV (GaN) to 6.2 eV (AlN). So, in theory, the cutoff wavelengths of a UV detector developed with intrinsic III-V materials can vary continuously between 365 nm and 200 nm.

The most attractive application in the group of III-nitride semiconductor (III–V) materials is for use in blue and UV photoelectric products [8,17,21,22,104,105,106]. However, a high density of dislocations and other structural defects hinder their use. Perfect crystallization of III–V nitride nanowires demonstrates their remarkable advantage in overcoming these problems, compared to the inherent poor crystallographic quality of bidimensional (2D) layers in this material family [106].

GaN is the most popular semiconductor in the III-nitride material family [17,107,108]. Li et al. [109] fabricated nanowire-based UV photodetectors with active parts made of GaN nanowire and electrodes made of Ag nanowire networks. Via a proper thermal annealing process, the dark current and response time of the photodetector showed considerable improvement under air and vacuum conductions. Verheij et al. [110] processed radiation sensors based on GaN microwires and demonstrated capability to detect UV light and protons. The performed opto-electrical characterization revealed a fast response to irradiation with UV light. Wang et al. [111] used a nonpolar a-axial GaN nanowire constructed for an MSM symmetrical Schottky contact device for use in a visible-blind UV detector. Without any surface or composition modifications, the fabricated device demonstrated superior performance, with sensitivity as high as 10^4^ A/W and external quantum efficiency of up to 10^5^, as well as an ultrafast response speed of less than 26 ms.

**Figure 12:**
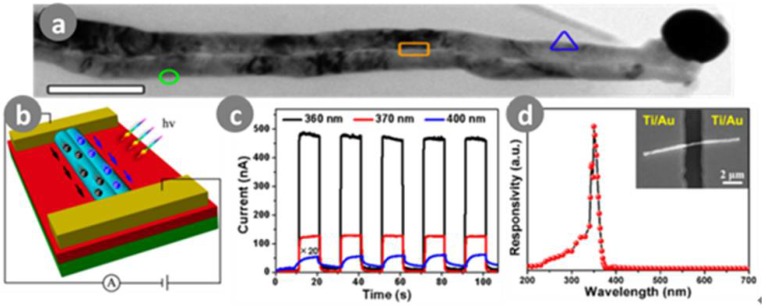
(**a**) TEM images of an single bicrystalline GaN nanowire with Ag catalyst terminated at the tip end; (**b**) Schematic diagram of a UV-A photodetector; (**c**) Under different irradiation wavelengths from 360 to 400 nm and a 5 V bias voltage and a power density of 6.41 W/cm^2^; (**d**) Photoresponse curves of bicrystalline GaN nanowire UV-A photodetector as a different wavelengths ranging from 200 to 700 nm, which is measured at a bias of 3 V. Ref. [113] Copyright 2017, American Chemical Society.

Zhang et al. [112] fabricated a GaN nanowire photodetector with Pt nanoparticle modification. Great UV photoresponse was achieved; the responsivity and EQE were 6.39 × 10^4^ A/W and 2.24 × 10^7^%, respectively. The same year, this team improved the photoresponsivity to 1.74 × 10^7^ A/W and the EQE to 6.08 × 10^9^% [113]. They prepared a photodetector based on individual bicrystalline GaN nanowires, as shown in Figure 12. This also demonstrated a fast photoresponse time (144 ms), high wavelength selectivity (UV-A light response only), and a very large on/off ratio of more than two orders.

**Figure 13:**
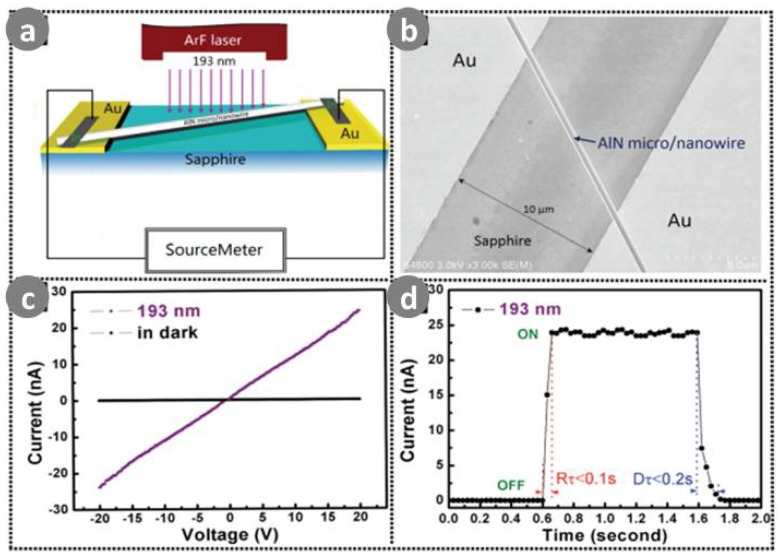
(**a**) Schematic presentation and; (**b**) representative SEM image of as-prepared AlN micro/nanowire-based photodetector; (**c**) *I*–*V* characteristicsof AlN micro/nanowire-based photodetector illuminated under a light of 193 nm (with an average power density of 1 W cm^−2^) and under dark condition; (**d**) Time-dependent response of the device measured under air environment at room temperature applied a bias of 20 V. Ref. [106] Copyright 2015, Wiley.

Specifically, AlN is a direct-gap semiconductor and its forbidden band is the widest among the III–V semiconductor materials [104]. This makes it an ideal preparative material for DUV/UV photoelectronic devices, and also as a new generation of information materials. Liu et al. [114] prepared ultra-long AlN nanowire arrays by CVD. The individual ultra-long AlN nanowires exhibited a clear photoconductive effect under the 325 nm UV wavelength. It was found that they had a fast response speed, high photocurrent response and reproductive working performance in the air environment. Zheng et al. [106] presented a two-step physical vapor transport method to grow high-quality AlN micro/nanowires. These were used to fabricate a VUV-sensitive photodetector with an ultra-short cutoff wavelength (193 nm), and fast photoresponse speed (<0.1 s) and recovery time (<0.2 s), as shown in Figure 13. The AlN micro/nanowire VUV photodetector showed a remarkable photocurrent increase for *λ* < 200 nm, and its response speed was 1–2 orders of magnitude faster than the corresponding values of other reported AlN thin-film photodetectors.

### 3.3. SiC

SiC has different polytypes or atomic arrangements. Commercially, the most common ones are 3C-SiC, 4H-SiC and, sometimes, 6H-SiC. The latter two have hexagonal (wurtzite) structures, while 3C-SiC has a cubic zinc-blende structure. All these polytypes consist of 50% Si and 50% C. The difference between the hexagonal polytype is the stacking order; they are not purely hexagonal. Atomic layers in the crystal are twisted in a certain sequence, and the sequence is shorter for 4H-SiC than 6H-SiC. Some material properties of the polytypes are listed in Table 1.

SiC is a good candidate for visible-blind UV photodetectors in high temperature, high power, and radiation-resistant applications, because of its wide band gap (2.36–3.05 eV), high breakdown electric field, high thermal conductivity, superior mechanical strength and excellent chemical stability. Accordingly, SiC-based devices can work in the harshest environments, including gaseous ones [48]. Benefiting from a large surface-to-volume ratio, long Debye length, low cost and relatively mature material technology [16], SiC nanowires can play an important role in UV detectors.

Peng et al. [48] prepared SiC nanowires by pyrolyzing a polymer precursor by a CVD route with ferrocene used as a catalyst. They fabricated UV-induced sensors based on a single such SiC nanowire. Three kinds of UV sensors based on single SiC nanowires were fabricated: with Schottky contacts, *p-n* junction contacts, and ohmic contacts, to study dark current and photocurrent *I-V* characteristics, photoresponse and time response properties. The results showed that the contacts between the electrode and the nanowires are important to a photodetector, the properties of which would be totally different if a different electrode fabrication process was used; for example, one which produced a different photoresponse, photocurrent, barrier and decay time. Teker [115] prepared single CVD-grown SiC nanowires. Under 254 nm UV light at a 2 V bias, a significant, positive, and fast photocurrent response was exhibited and showed great reversibility and recovery in photoconductance. The mechanism demonstrated that complete depletion of the space charge layer of the SiC nanowire enhanced the surface recombination of photoexcited electron-hole pairs.

### 3.4. Conclusions

Table 2 summarizes existing studies on nanowire-based UV detectors. Metal oxide semiconductors include ZnO, TiO_2_, SnO_2_ and so on, all of which are very good candidates for use in photonics devices working in the UV range. Metal oxide-based UV photodetectors show good responsivity, high UV contrast ratios, high speed and low noise characteristics. In theory, the cutoff wavelengths of UV detectors developed with the intrinsic material of III–V group semiconductors can be continuously varied from 365 nm to 200 nm. Specifically, the band gap width of AlN can reach 6.20 eV, making this an ideal preparative material for DUV/UV photoelectronic devices. SiC is a very good candidate for visible-blind UV photodetectors used in high temperature, high power, and radiation-resistant applications, because of its wide band gap, high breakdown electric field, high thermal conductivity and low thermal expansion properties.

## 4. New Types of UV Dtectors

### 4.1. Hybrid Nanostructure UV Detectors

**Figure 14:**
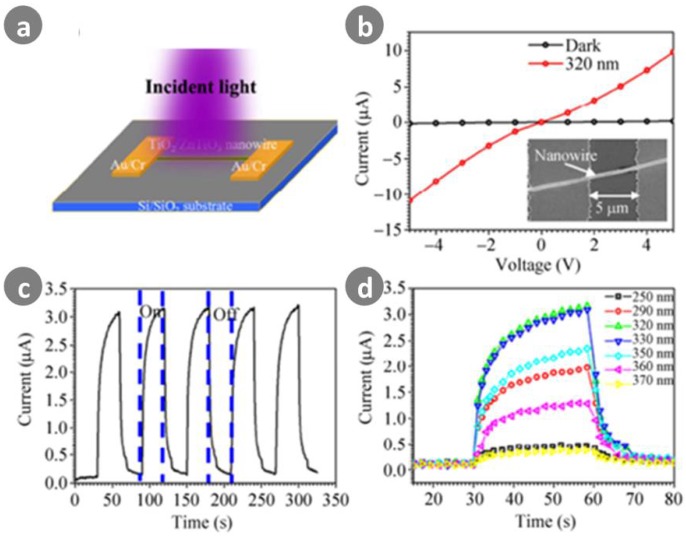
(**a**) Schematic of single TiO_2_-ZnTiO_3_ nanowire-based photodetector; (**b**) *I–V* curves of a single nanowire-based photodetector; inset is a corresponding SEM image of the device; (**c**) Reproducible on/off switching illuminated by 320 nm light with intensity of 186.2 μW/cm^2^ at a bias of 2 V; (**d**) Spectroscopic photoresponse of the photodetector under UV illumination under light with varying wavelengths (250 nm: 208.5 μW/cm^2^, 290 nm: 185.5 μW/cm^2^, 320 nm: 186.2 μW/cm^2^, 330 nm: 191.2 μW/cm^2^, 350 nm: 176.1 μW/cm^2^, 360 nm: 180.9 μW/cm^2^, 370 nm: 202 μW/cm^2^) at a bias of 2 V. Ref. [116] Copyright 2015, Tsinhua University Press.

Hybrid nanostructures are considered as most promising sensitive photodetection materials because they offer not only high photoconductive gain and the property benefits of 1D nanostructures, but also the added benefit of multifunctional or new properties arising from the synergistic effects of combining heterojunction materials. Chong et al. [116] successfully developed TiO_2_-ZnTiO_3_ heterojunction nanowire-based photodetectors and used them to detect solar-blind UV light. As shown in Figure 14, further analysis indicated that the rich existence of grain boundaries within the TiO_2_-ZnTiO_3_ nanowire can greatly decrease the dark current and recombination of the electron-hole pairs, and thereby significantly increase the device’s photosensitivity, spectral responsivity (1.1 × 10^6^ A/W), and external quantum efficiency (4.3 × 10^8^%).

Recently, 1D core/shell nanostructures have been utilized in photodetectors because they have a larger specific surface area, better light-trapping effect, and longer carrier lifetime compared to pristine nanostructures. For example, self-assembled nanowires containing InGaN/GaN core/shell quantum wells were synthesized in the middle of a radial *p-n* junction by catalyst-free metal-organic vapor phase epitaxy [117]. With the same method, Zhang et al. [118] fabricated single *p-n* junction nanowire photodetectors containing 30 non-polar radial InGaN/GaN quantum well system on sapphire substrates. Rai et al. [119] fabricated a high-performance UV detector on a fully wide bandgap ZnO/ZnS heterojunction core/shell nanowire array. Analogously, Park et al. [120] fabricated multiple networked ZnO-ZnS core/shell nanowire photodetectors.

By adopting a vacuum-free low-cost chemical fabrication route, Dao et al. [121] fabricated a high-sensitivity UV photodetector composed of a *p-n* heterojunction core/shell nanowire architecture using *n*-type ZnO and *p*-type TiO_2_. The device operated as a model *p-n* heterojunction with a linear response and sensitivity larger than 250 A/W (5 V reverse bias, UV illumination at 373 nm). Shin et al. [122] reported the formation of a *p-n* junction through the use of CuO/Cu_1−x_In_x_O core/shell nanowire structures for application in photodetectors. The fabricated core/shell *p-n* junction exhibited a photoresponsivity of 0.045 A/W at 374 nm.

### 4.2. Self-Powered UV Detectors

Conventional UV detectors generally need an external bias voltage as a driving force to generate photocurrent. This limits the size of the nanodevice and its independent working system. Thus, a new type of UV photodetector which operates without any power supply, namely, *self-powered photodetectors*, have drawn much attention. Self-powered photodetectors work at zero bias without consuming external power, hence they are more portable and adaptable than conventional photodetectors.

Generally, such self-powered photodetectors fall into two major classes according to energy conversion. One class is based on the photovoltaic effect, which can be directly driven by an optical signal, and convert light energy into electric energy through the photoelectron excitation process. The other class contains an integrated energy unit, which transforms UV radiation into electrical and chemical energy. These devices usually operate by a combination of the photoelectric and photoelectrochemical (PEC) effects.

The photovoltaic effect has huge potential for exploitation in novel self-powered UV photodetectors containing *p-n* junctions, heterojunctions, or Schottky junctions. Yang et al. [123] demonstrated the first n-ZnO nanowire/polyaniline/ZnGa_2_O_4_ (a *p-n* and a type-II heterojunction)-based photodetector devices that were self-powered and had sensitivity to UV light at different wavelengths with complete current reversal capabilities. The devices also showed remarkable environmental stability. Similarly, self-powered and rapid-response p-NiO/n-ZnO nanowire UV photodetectors were fabricated and investigated using Al_2_O_3_ as an interface modification layer by Chen et al. [124]. A responsivity of 1.4 mA/W was achieved under 380 nm UV irradiation (0.36 mW/cm^2^) at zero bias, and the response time of the device was less than 0.04 s.

Recently, progress has been made in developing a novel PEC-type self-powered photodetector, as exemplified by the work of Zhang et al. They synthesized ZnO/Cu_2_O branched heterojunction arrays for application in photoelectrochemical-type self-powered UV-visible photodetectors [125]. At zero bias, the photodetectors based on ZnO/Cu_2_O nanowire/electrolyte heterojunctions exhibited responsivities of 19.3 mA/W and 8.2 mA/W for UV and visible light, respectively, with a rise time of 0.14 s and a decay time of 0.36 s. They then enhanced the self-powered photoresponse performance of the ZnO nanowire arrays/Cu_2_O photoanodes by introducing graphene interlayers [126]. Their self-powered photodetectors based on ZnO nanowire arrays/graphene/Cu_2_O had responsivities of 21.2 mA/W and 17.1 mA/W for ultraviolet and visible light, respectively, and fast rise and decay times (0.6 ms). They also designed and fabricated photoelectrochemical-type self-powered photodetectors based on patterned ZnO/CdS nanowire arrays [127].

**Figure 15:**
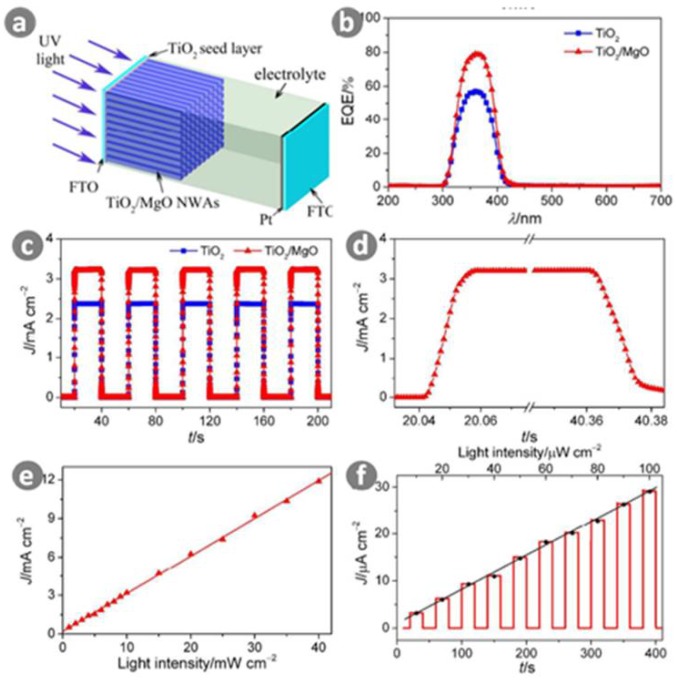
(**a**) Device structure diagram of a photoelectrochemical self-powered UV detector; (**b**) EQE spectra of TiO_2_ and TiO_2_/MgO nanowires based UV detectors; (**c**) Photocurrent responses of TiO_2_ and TiO_2_/MgO nanowires based UV detectors under on/off radiation of 10 mW/cm^2^ UV light illumination (*λ* = 365 nm); (**d**) Enlarged rising and decaying edges of the photocurrent response for the TiO_2_/MgO nanowires based UV detectors; (**e**) *J* as a function of the incident UV light intensity from 1 to 40 mW/cm^2^ for the TiO_2_/MgO nanowires based UV detectors; (**f**) Photocurrent responses of the TiO_2_/MgO nanowires based UV detectors under on/off radiation of low UV light density from 10 to 100 μW/cm^2^. Ref. [128] Copyright 2016, Royal Society of Chemistry.

Ni et al. [128] fabricated a photoelectrochemical self-powered UV photodetector using heterostructured TiO_2_/MgO nanowire arrays as the photoanode. As shown in Figure 15, their UV photodetector exhibited high photocurrent density and open-circuit voltage, which was ascribed to a lower charge recombination process at the interfaces of the TiO_2_/MgO nanowire arrays and the electrolyte. More importantly, their UV photodetector based on TiO_2_/MgO nanowire arrays exhibited a remarkable responsivity of 0.233 A/W at 365 nm, a high on/off ratio of 14,879, a fast rise time of 0.010 s, and a decay time of 0.011 s under UV irradiance, together with excellent spectral selectivity and linear optical signal response.

### 4.3. Flexible UV Detectors

Recently, flexible devices have become more prevalent with the aim of developing portable, wearable, lightweight and implantable optoelectronic devices. This also offers numerous opportunities for the development of next-generation UV detectors. Up to now, research on flexible UV detectors has been carried out by many groups. A flexible UV detector based on SnO_2_ nanowire arrays was fabricated by Kim et al. [129]. They demonstrated excellent UV sensing characteristics, demonstrating an average photosensitivity of ∼10^5^ and a photoconductive gain of ∼10^6^ under very low UV power intensities of 0.02–0.04 mW/cm^2^. Device performance did not deteriorate with prestrain of up to 23% induced by radial deformation. Park et al. [130] proposed a new fabrication method; that of roll-to-roll processed ZnO nanowire-based UV photodetectors containing flexible film. A maximum photocurrent of 62.1 mA, response time of 9.1 s, and recovery time of 56 s were obtained at a bias voltage of 10 V under UV irradiation of 127 μW/cm^2^. Patel et al. [131] demonstrated a transparent and flexible photodetector using an Ag nanowire-network. Under UV illumination, remarkable performance was obtained, including quick response (rise time = 0.987 ms, fall time = 2.49 ms) and ultrahigh responsivity (1.46 × 10^4^ A/W).

**Figure 16:**
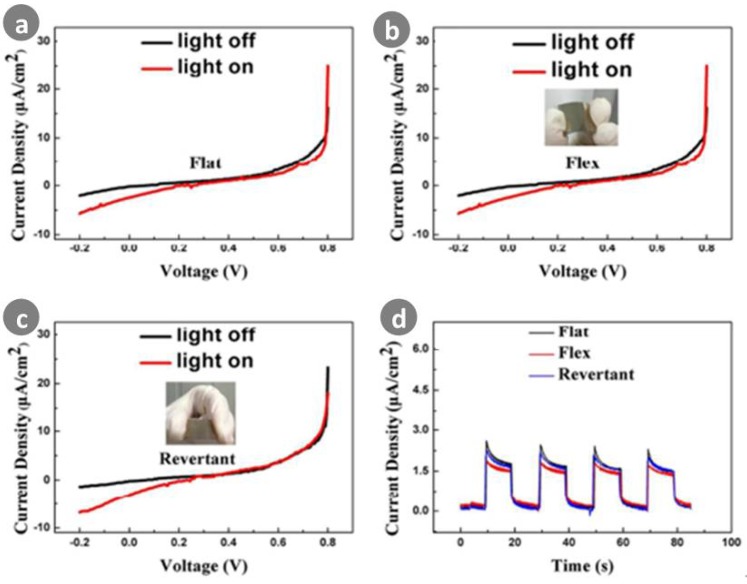
(**a**) I–V characteristics of ZnO nanowires based detector (sample 1) working in the original status in darkness and under the illumination of 60 µW/cm^2^ of UV light (*λ* = 365 nm); (**b**) I–V characteristics of ZnO nanowires based detector working in the bending status; (**c**) I–V characteristics of ZnO nanowires based detector working in the status after the removal of bending force; (**d**) Photocurrent response of ZnO nanowires based device in three working conditions (flat, flex, revertant) under on/off radiation of 60 µW/cm^2^ of UV light (*λ* = 365 nm). Ref. [132]. Copyright 2016, Royal Society of Chemistry.

Zeng et al. [132] demonstrated a facile method for growing ZnO nanowires on polyethylene terephthalate (PET) substrates, and fabricated a flexible, self-powered UV detector. As shown in Figure 16, the assembled UV detector showed good wear-ability and flexibility. By depositing certain-sized Ag nanoparticles onto ZnO nanowires, the responsivity of the fabricated device was improved by 44%. Such enhancement was attributed to the modified structure, which facilitates light absorption.

## 5. Summary and Outlook

This review summarized the various types of wide bandgap semiconductor nanowires for use in different types of UV detectors. Photoconductive UV detectors have simple structures, easy process control and high internal gain advantages, but have slow response speeds and large dark currents. In comparison, photovoltaic detectors provide advantages in terms of both sensitivity and operational speed. The photovoltaic detectors including Schottky photodiodes, *p-n* and *p-i-n* junction photodiodes, MSM photodetectors are discussed. Each of these different types of devices has certain advantages for UV detection, depending on the particular applications. This article also reviewed some of the important materials with nanowire structures, and their UV-detecting properties. The new types of photodetectors have gradually prompted the development of modern UV photodetectors. Fabrication of UV photodetectors based on heterostructures or nanocomposite structures will be very valuable, due to their multifunctional and property-tuning potential. Self-powered photodetectors will not only greatly enhance the adaptability of the devices, but also greatly reduce the size and weight of the systems. With the flourishing development of flexible photodetectors, great progress in portable and wearable devices will be made.

Of course, there are other UV detectors that are manufactured with infrequently-used materials, or commonly-used materials subject to unique processes; these are also worthy of consideration. Creation of a deep UV detector with high sensitivity, low noise, strong resistance to radiation, and high stability and reliability, would greatly promote the national economy and the development of aerospace enterprises.

## Figures and Tables

**Table 1:** The properties of SiC polytypes.

SiCPolytype	Bandgap at 300 K [eV]	Cut Off Wavelength [nm]	Electron Mobility [cm^2^V^−1^s^−1^]	Hole Mobility [cm^2^V^−1^s^−1^]
3C	2.36	525	<800	<320
4H	3.23	384	<900	<120
6H	3.05	407	<400	<90

**Table 2:** The important parameters of UV detectors reported.

Materials of Nanowires	Light (nm)	Photocurrent (A)	Dark Current (A)	Responsivity (A/W)	EQE/Gain	Response Time (s)	Reference
ZnO	370	-	-	10^5^	10^8^%		[70]
ZnO	365	1.79 × 10^−4^	-	-	2420%	3.9	[71]
ZnO	365	-	-	-	-	0.45	[72]
ZnO/Ag	365	-	-	4.91 × 10^6^	1.67 × 10^9^%	-	[73]
ZnO/Graphene	-	6.3 × 10^−4^	-	1.62	-	0.3	[75]
ZnO/Graphene	-	-	-	-	2490.8%	9.5	[76]
TiO_2_ (FTO)	350	1.52 × 10^−5^	3.6 × 10^−^^9^	0.17	60.7%		[81]
(ITO)		3.37 × 10^−6^	2.36 × 10^−^^8^	0.38	13.4%	-	
TiO_2_	330	-	1.9 × 10^−9^	5.68 × 10^−1^	-	-	[82]
TiO_2_	-	1.67 × 10^−9^	2 × 10^−12^	-	-	0.4	[83]
TiO_2_	375	-	-	90	-	5	[84]
SnO_2_	320	2.1 × 10^−6^	1.94 ×10^−8^	-	1.32 × 10^9^%	-	[91]
SnO_2_	325	-	-	-	2.5 × 10^7^%	-	[92]
SnO_2_	365	2.3 × 10^−6^	2.1 × 10^−^^7^	-	-	0.1	[93]
SnO_2_	250	-	-	1.2 × 10^7^	6.0 × 10^9^%	-	[94]
VO_2_	360–400	-	-	7.07 × 10^3^	2.4 × 10^10^%	0.126	[95]
*β*-Ga_2_O_3_	250	-	-	3.77 × 10^2^	2 × 10^5^%	1.2 × 10^−6^	[96]
In_2_O_3_	405	2.17 × 10^−2^	4.5 × 10^−4^	4.8 × 10^6^	1.46 × 10^9^%	3	[97]
K_2_Nb_8_O_21_	320	1.35 × 10^−11^	1.2 × 10^−12^	2.53	982%	<0.3	[98]
InGaO_3_(ZnO)	350	4.71 × 10^−7^	4.3 × 10^−9^	5.3 × 10^4^	1.9 × 10^9^%	0.3	[99]
ZnGa_2_O_4_	350	5.2 × 10^−8^	4 × 10^−10^	3.174	1.1 × 10^6^%	15	[100]
Zn_2_GeO_4_	260	-	1 × 10^−9^	5.11 × 10^3^	2.45 × 10^8^%	0.01	[101]
GaN	325	10^−8^	-	2.2 × 10^4^	3.2 × 10^7^%	<0.026	[111]
GaN/Pt	380	-	-	6.39 × 10^4^	2.24 × 10^7^%	1.1	[112]
GaN	320–400	-	-	1.74 × 10^7^	6.08 × 10^9^%	0.144	[113]
AlN	325	-	-	2.7 × 10^6^	-	0.001	[114]
AlN	193	2.4 × 10^−8^	1 × 10^−14^	0.39	254%	<0.1	[106]
SiC (Schottky)	-	4.3 × 10^−^^6^	4.3 × 10^−^^8^	-	-		[48]
SiC	254	-	-	-	-	3	[115]
